# Personalized cancer medicine: next steps in the genomic era

**DOI:** 10.1007/s13402-015-0221-0

**Published:** 2015-02-27

**Authors:** S. Derks, B. Diosdado

**Affiliations:** 1 Department of Medical Oncology, Dana-Farber Cancer Institute, Boston, MA USA; 2 VU University Medical Center, Amsterdam, The Netherlands; 3 Department of Pathology, VU University Medical Center, Amsterdam, The Netherlands

In recent years massive amounts of genomic information characterizing the majority of human cancers have become available, which has profoundly changed our perception of the complexity of this disease. Huge efforts by The Cancer Genome Atlas (TCGA) and the International Cancer Genome Consortium (ICGC) projects have reshaped the landscape of mutations, transcription signatures, epigenetic profiles and copy number changes in cancer [1, 2]. Integrating this (epi-)genomic and transcriptomic information with clinical data has led to a first glimpse on inter- and intra-tumor heterogeneity in relation to diagnosis, prognosis and response to therapy, and to the identification of novel targetable molecular alterations [3]. We are now facing a decennium in which we will be able to integrate the currently used TNM system with novel molecular markers to better classify and stage tumors, to better stratify patients and to better use molecular markers to develop biology-based individualized treatment options. In this special issue of *Cellular Oncology* some key recent developments and points of concern in the field of personalized cancer care are reviewed, including which steps still need to be taken before the final implementation of personalized cancer medicine becomes a clinical reality.

One of the major goals in the coming years will be the identification of biological driver events in cancers for which no targeted therapeutic options are available yet. Currently, still the majority of patients with cancer are treated using standard therapies (i.e., surgery, radiotherapy, chemotherapy). This is mostly due to the fact that targetable molecular alterations have so far only been identified in a handful of cancer types and in a minority of patients. Unsupervised molecular classification of human cancers is increasingly leading to the identification of driver molecular pathways and their associated clinical features. Salazar and colleagues [4] highlight in this special issue how the integration of (epi-)genomic data in an unsupervised manner can re-define the above mentioned molecular heterogeneity present in human cancers at a deeper level, and how this integration can reveal cross-talk between molecular pathways. In this manner, colon, lung and breast cancers have e.g. been re-classified into molecular subgroups based on their biological characteristics and, concomitantly, their newly identified targetable pathways. As a corollary of this novel information, possible mechanisms of resistance to targeted therapies have also been proposed. While so far most studies have primarily focused on genomic (exomic) data, it is becoming increasingly clear that not only mutations or copy number changes are underlying the immense complexity of human cancers. In this special issue Poliseno and colleagues [5] nicely illustrate the emerging role and clinical significance of a large group of regulatory RNAs, with special emphasis on long-non-coding RNAs, pseudogenes and circular RNAs. The authors illustrate via several examples the profound role that these molecules can play in diverse human cancers, their potential as diagnostic and prognostic markers, and their dual role as both therapeutic agents and targets.

Several technological developments have facilitated the in-depth detection of large numbers of molecular alterations in a single tumor in a relatively time- and cost-effective way, such that these technologies can be implemented in both routine research and clinical practice [6]. The clinical interpretation of these vast amounts of information is, however, still challenging. Van Neste et al. [7] describe in this special issue the importance of appropriate bioinformatics tools to make this information manageable, interpretable and actionable, and they also highlight the work that still needs to be done before large genomic screens can be implemented in routine patient care. Specifically, it is still a major challenge to effectively separate driver from passenger events and to delineate genetic and epigenetic alterations in the same pathway in an integrated fashion. With the growing amount of (epi-)genomic information that is generated per patient, the main challenges will be to efficiently mine and combine independent data sets and to make these data sets meaningful for a broader audience.

Another challenge that lies at the basis of both future research and the translation and clinical implementation of its findings into tools for personalized medicine, is the organization of health care itself. The logistics of assembling and handling samples is another major issue. To this end, Nuciforo and colleagues [8] emphasize in this special issue how sample collection, storage and processing, biological heterogeneity and study design can profoundly influence the results and the interpretation of the molecular findings in the context of routine clinical diagnostics and trials.

In order to circumvent invasive methods multiple initiatives are currently ongoing, including studies aimed at using circulating tumor cells or tumor DNAs or, alternatively, molecular imaging. In this special issue Mammatas et al. [9] introduce the potential role of molecular imaging in personalized cancer medicine and provide an overview of recent developments in this field. They emphasize that molecular imaging is a non-invasive method that can be extremely useful to study a tumor’s heterogeneity and response to therapy. Response to therapy does not only depend on the (ectopic) expression of certain proteins and drug uptake by the tumor, but can also be influenced by germline variants in genes involved in drug metabolism and transport, a field of study named pharmacogenomics. Testing for these variants may result in increased drug efficacy through adapting drug doses to personal needs. In this special issue Pesenti et al. [10] review recent developments in the field of pharmacogenomics and provide an overview of germline variants that can be tested in clinical practice and, thus, can be employed to contribute to personalized cancer medicine.

While tremendous efforts in recent years have paved the way for the next step in personalized biology-driven cancer medicine in the genomic era, major challenges are still lying ahead of us. Once these challenges have been turned into opportunities for the application of diagnostic tests and personalized treatment options, the prospect glimmers on the horizon for the medical community to offer the best practice to cancer patients.
